# Medical Education and Medical Reform

**Published:** 1839-07

**Authors:** 


### PART FOURTH.

### Medical Intelligence.

### MEDICAL EDUCATION AND MEDICAL REFORM.

### I. *Extracts from the "Regulations of the University of London, on the subject of Examinations for Degrees in Medicine."*

#### 1. MATRICULATION EXAMINATION.

No candidate shall be admitted to the matriculation examination unless he have produced a certificate showing that he has completed his sixteenth year.

A fee of two pounds shall be paid at matriculation. No candidate shall be admitted to the examination unless he have previously paid this fee to the registrar; and if he fail to pass the examination, the fee shall be returned to him.

The examination shall be conducted by means of printed papers; but the examiners shall not be precluded from putting any *vivâ voce* questions upon the written answers of the candidates, when they appear to require explanation.

Candidates for the matriculation examination shall be examined in the following subjects:

##### MATHEMATICS.

*Arithmetic and Algebra.*—The ordinary rules of arithmetic. Vulgar and decimal fractions. Extraction of the square root. Addition, subtraction, multiplication, and division of algebraical quantities. Proportion. Arithmetical and geometrical progression. Simple equations.

*Geometry.*—The first book of Euclid.

##### NATURAL PHILOSOPHY.

*Mechanics.*—Explain the composition and resolution of statical forces. Describe the simple machines (mechanical powers), and state the ratio of the power to the weight in each. Define the centre of gravity. Give the general laws of motion, and describe the chief experiments by which they may be illustrated. State the law of the motion of falling bodies.

*Hydrostatics, Hydraulics, and Pneumatics.*—Explain the pressure of liquids and gases, its equal diffusion, and variation with the depth. Define specific gravity, and show how the specific gravity of bodies may be ascertained. Describe and explain the barometer, the siphon, the common pump and forcing pump, and the air-pump.

*Acoustics.*—Describe the nature of sound.

*Optics.*—State the laws of reflexion and refraction. Explain the formation of images by simple lenses.

##### CHEMISTRY.

The component parts of the atmosphere and of water. The general characters of the different groups of elementary bodies, namely, of the supporters of combustion, the combustibles, and the metals. The influence of heat upon the bulk and states of matter.

##### NATURAL HISTORY.

*Botany.*—The characters and differences of the natural classes and principal orders of phanerogamous plants belonging to the Flora of Europe, in the botanical classification of De Candolle.

*Zoology.*—The characters of the primary divisions of the animal kingdom, and of the classes and orders of the vertebrate sub-kingdom, according to the system of Cuvier.

##### CLASSICS.

*The Greek and Latin Languages.*—One Greek and one Latin subject, to be selected one year previously by the Committee of the Faculty of Arts from the works of the following authors: Homer, one book; Xenophon, one book; Virgil, one book of the Georgics, or the sixth book of the Æneid; Horace, one book of the Odes; Sallust, the Conspiracy of Cataline, or the war with Jugurtha; Cæsar, the Civil War, or the fifth and sixth books of the Gallic War; Livy, one book; the treatises De Senectute and De Amicitiâ, or two of the shorter, or one of the longer orations.

*The English Language.*—The grammatical structure of the language. Proficiency in composition will be judged of by the style of answers generally.

*Outlines of History and Geography.*—History of England to the end of the seventeenth century. The papers in classics shall contain questions in history and geography.

EXAMINATION FOR HONOURS.

Any candidate who has passed, and has produced a certificate showing that he has not completed his nineteenth year, may be examined for honours in mathematics and natural philosophy, and also in classics.

In determining the relative position of candidates, the examiners shall have regard to the proficiency in mathematics evinced by the candidates at the matriculation examination. The examiners shall publish a list of the candidates in the order of proficiency; and candidates shall be bracketed together, unless the examiners are of opinion that there is a clear difference between them.*

If in the opinion of the examiners any candidate shall possess sufficient merit, the candidate who shall distinguish himself the most in mathematics and natural philosophy, and the candidate who shall distinguish himself the most in classics, shall each receive an exhibition of thirty pounds per annum for the next two years, if continuing during that period students at one of the institutions in connexion with this University.

### 2. BACHELOR OF MEDICINE.

Candidates for the Degree of Bachelor of Medicine shall be required, 1. To have been engaged during four years in their professional studies at one or more of the institutions or schools recognized by this University. 2. To have spent one year at least of the four in one or more of the recognized institutions or schools in the United Kingdom. 3. To pass two examinations.

FIRST EXAMINATION.

No candidate shall be admitted to this examination unless he have produced certificates to the following effect: 1. Of having completed his nineteenth year. 2. Of having taken a degree in arts in this University, or in a University the degrees granted by which are recognized by the senate of this University, or of having passed the matriculation examination. 3. Of having been a student during two years at one or more of the medical institutions or schools recognized by this University, subsequently to having taken a degree in arts, or passed the matriculation examination. 4. Of having attended a course of lectures on each of four of the subjects in the following list: Descriptive and Surgical Anatomy; General Anatomy and Physiology; Comparative Anatomy; Pathological Anatomy; Chemistry; Botany; Materia Medica and Pharmacy; General Pathology; General Therapeutics; Forensic Medicine; Hygiene; Midwifery and Diseases peculiar to Women and Infants; Surgery; Medicine. 5. Of having dissected during nine months. 6. Of having attended a course of Practical Chemistry, comprehending practical exercises in conducting the more important processes of general and pharmaceutical chemistry; in applying tests for discovering the adulteration of articles of the materia medica, and the presence and nature of poisons; and in the examination of Mineral Waters, Animal Secretions, Urinary Deposits, Calculi, &c. 7. Of having attended to Practical Pharmacy during a sufficient length of time to enable him to acquire a practical knowledge in the preparation of medicines.

* This regulation holds good for all the other examinations.—Ed.

The fee for this examination shall be five pounds. No candidate shall be admitted to the examination unless he have previously paid this fee to the registrar; and if he fail to pass the examination the fee shall be returned to him.

Candidates shall be examined in the following subjects: Anatomy; Physiology; Chemistry; Structural and Physiological Botany; Materia Medica and Pharmacy.

EXAMINATION FOR HONOURS.

Any candidate who has been placed in the first division at the first examination, and who has produced a certificate showing that he has not completed his twenty-second year, may be examined for honours in any or all of the following subjects: Anatomy and Physiology (candidates may illustrate their answers by sketching the parts they describe); Chemistry; Materia Medica and Pharmaceutical Chemistry.

The examinations shall take place in the week following the first examination. They shall be conducted by means of printed papers; but the examiners shall not be precluded from putting *vivâ voce* questions upon the written answers of the candidates when they appear to require explanation.

If in the opinion of the examiners sufficient merit be evinced, the candidate who shall distinguish himself the most in anatomy and physiology, the candidate who shall distinguish himself the most in chemistry, and the candidate who shall distinguish himself the most in materia medica and pharmaceutical chemistry, shall each receive an exhibition of thirty pounds per annum for the next two years.

Under the same circumstances the first and second candidates in each subject shall each receive a gold medal of the value of five pounds.

SECOND EXAMINATION.

No candidate shall be admitted to this examination within two academical years of the time of his passing the first examination, nor unless he have produced certificates to the following effect:

1. Of having passed the first examination.

2. Of having, subsequently to having passed the first examination, attended a course of lectures on each of two of the subjects comprehended in the list given under the head of the First Examination, and for which the candidate had not presented certificates at the first examination.

3. Of having, subsequently to having passed the first examination, dissected during six months.

4. Of having conducted at least six labours. Certificates on this subject will be received from any legally-qualified practitioner in medicine.

5. Of having attended the surgical practice of a recognized hospital or hospitals during twelve months, and lectures on clinical surgery.

6. Of having attended the medical practice of a recognized hospital or hospitals during other twelve months, and lectures on clinical medicine.

7. Of having, subsequently to the completion of his attendance on surgical and medical hospital practice, attended to practical medicine in a recognized hospital, infirmary, or dispensary, during six months. Certificates on this subject will be received from any legally-qualified practitioner having the care of the poor of a parish.

The candidate shall also produce a certificate of moral character from a teacher in the last school or institution at which he has studied, as far as the teacher's opportunity of knowledge has extended.

The fee for this examination shall be five pounds. No candidate shall be admitted to the examination unless he have previously paid this fee to the registrar; and if he fail to pass the examination, the fee shall be returned to him.

Candidates shall be examined in the following subjects:

Physiology: the papers in physiology shall include questions in comparative anatomy. General pathology, general therapeutics, hygiene; surgery; medicine; midwifery; forensic medicine.

EXAMINATION FOR HONOURS.

Any candidate who has been placed in the First Division at the second examination, and has produced a certificate showing that he has not completed his twenty-fifth year, may be examined for honours in any or all of the following subjects—Physiology and Comparative Anatomy (candidates may illustrate their answers by sketching the parts they describe); Surgery; Medicine; Midwifery; Structural and Physiological Botany.

The examinations shall take place in the week following the second examination. They shall be conducted by means of printed papers; but the examiners shall not be precluded from putting *vivâ voce* questions upon the written answers of the candidates when they appear to require explanation.

If in the opinion of the examiners sufficient merit be evinced, the candidate who shall distinguish himself the most in Physiology and Comparative Anatomy, the candidate who shall distinguish himself the most in Surgery, and the candidate who shall distinguish himself the most in Medicine, shall each receive an exhibition of fifty pounds per annum for the next two years, with the style of University Medical Scholar.

Under the same circumstances, the first and second candidates in each of the preceding subjects shall each receive a gold medal of the value of five pounds.

Under the same circumstances the candidate who shall distinguish himself the most in Midwifery, and the candidate who shall distinguish himself the most in Structural and Physiological Botany, shall each receive a gold medal of the value of five pounds.

### 3. DOCTOR OF MEDICINE.

No candidate shall be admitted to this examination unless he have produced certificates to the following effect: 1. Of having taken the degree of Bachelor of Medicine in this University, or a degree in Medicine or in Surgery at a University the degrees granted by which are recognized by the Senate of this University. Those candidates who have not taken the degree in this University shall produce a certificate of having completed their twenty-third year. 2. Of having attended, subsequently to having taken one of the above degrees in Medicine—*a*. To Clinical or Practical Medicine during two years in a hospital or medical institution recognized by this University. *b*. Or, to Clinical or Practical Medicine during one year in a hospital or medical institution recognized by this University, and of having been engaged during three years in the practice of his profession. *c*. Or, if he have taken the degree of Bachelor of Medicine in this University, of having been engaged during five years in the practice of his profession. One year of attendance on Clinical or Practical Medicine, or two years of practice, will be dispensed with in the case of those candidates who at the second examination have been placed in the First Division. 3. Of moral character, signed by two persons of respectability.

The fee for the degree of Doctor of Medicine shall be ten pounds. No candidate shall be admitted to the examination unless he have previously paid this fee to the registrar; and if he fail to pass the examination the fee shall be returned to him. The examination shall be conducted by means of printed papers and *vivâ voce* interrogation. Candidates shall be examined in the following subjects: Elements of Intellectual Philosophy, Logic, and Moral Philosophy; Medicine.

If in the opinion of the examiners sufficient merit be evinced, the author of the best commentary on the Case in Medicine, the author of the best commentary on the Case in Surgery, and the author of the best commentary on the Case in Midwifery, shall each receive a gold medal of the value of five pounds.

Any candidate may present a thesis on a subject of his own choice. If in the opinion of the examiners sufficient merit be evinced, a gold medal, of the value of ten pounds, shall be given to the author of the best thesis. The examiners shall not be precluded from examining the author on the subject of his thesis.

#### EXAMINATION FOR HONOURS.

Any candidate who has been placed in the First Division may be examined for honours in any or all of the following subjects: Surgery, Medicine, Midwifery.

The examinations shall be conducted by means of printed papers; but the examiners shall not be precluded from putting *vivâ voce* questions upon the written answers of the candidates when they appear to require explanation.

If in the opinion of the examiners sufficient merit be evinced, the first candidate in each subject shall receive a gold medal of the value of five pounds.

### INSTITUTIONS AND SCHOOLS.

No medical institution or school shall be recognized by the Senate of this University which does not possess ample means of illustrating the instruction given at it.

*Regulations relating to Students who commenced their Medical Studies in or before January,* 1839, *to continue in force until the year* 1842.

### BACHELOR OF MEDICINE.

Previously to the year 1842, candidates who have been engaged during two years in their professional studies shall be admitted to the first examination for the degree of Bachelor of Medicine on producing certificates to the following effect: 1. Of having been engaged during two years in their professional studies. 2. Of having attended a course of lectures on each of four of the subjects comprehended in the list given under the head of the *First* Examination. 3. Of having dissected during nine months. 4. Of having attended to Practical Pharmacy during a sufficient length of time to enable them to acquire a practical knowledge in the preparation of medicines.

Candidates shall be admitted to the second examination at the expiration of two years after the first examination, on producing the certificates required at that examination.

Previously to the year 1842, candidates who have been engaged during four years in their professional studies shall be admitted to the second examination for the degree of Bachelor of Medicine, on producing certificates to the following effect: 1. Of having been engaged during four years in their professional studies. 2. Of having passed the first examination. 3. Of having attended a course of lectures on each of two of the subjects comprehended in the list given under the head of the *First* Examination. 4. Of having dissected during twelve months. 5. Of having attended to practical pharmacy during a sufficient length of time to enable the pupil to acquire a practical knowledge in the preparation of medicines. 6. Of having conducted at least six labours. 7. Of having attended the surgical practice of a recognized hospital or hospitals during twelve months. 8. Of having attended the medical practice of a recognized hospital or hospitals during other twelve months. 9. Of having completed the twenty-second year of their age. 10. Of moral character from a teacher in the last school, or institution at which they have studied, as far as the teacher's opportunity of knowledge has extended.

Candidates who have not taken a degree in Arts, or passed the Matriculation Examination in this University, will be required to translate a portion of Celsus *de Re Medica.*

*Regulations relating to Practitioners in Medicine or Surgery desirous of obtaining Degrees in Medicine.*

### BACHELOR OF MEDICINE.

Candidates shall be admitted to the two examinations for the degree of Bachelor of Medicine on producing certificates to the following effect: 1. Of having been admitted, prior to the year 1840, members of one of the legally-constituted bodies in the United Kingdom for licensing practitioners in medicine or surgery. 2. Of having received a part of their education at a recognized institution or school, as required by the charter of the University. 3. Of moral character, signed by two persons of respectability.

### DOCTOR OF MEDICINE.

Candidates who have been engaged during five years in the practice of their profession shall be admitted to the examination for this degree on producing certificates to the following effect: 1. Of having been engaged during five years in the practice of their profession. 2. Of having taken the degree of Bachelor of Medicine in this University.

---

II. *Extracts from "Propositions relative to the Education and Privileges of Practitioners in the several Branches of Medicine, and of Chemists and Druggists: agreed on by the Medical and Surgical Professors in the University, the Royal College of Physicians, and the Royal College of Surgeons of Edinburgh. March,* 1839."

1. That the legislature ought to fix a minimum course of education, without certificates of having accomplished which, no one should be admissible to examination for a licence to practise any of the branches of medicine.

2. That without such a licence, no one should be eligible to hold a medical or surgical appointment in any public institution.

3. That no person ought to obtain a licence entitling him to act as a general medical practitioner, who has not received a competent education in literature and science, studied in a recognized school of medicine or surgery, and undergone examination before a competent board or boards, on all the branches of medical education mentioned in the curriculum hereinafter specified.

4. That the degrees or licences granted by all public institutions which have heretofore been engaged in regulating the education, and ascertaining the qualifications, of those intended for the medical profession (or by such new boards as it may be found expedient to establish for the same purposes), should confer the right of acting as general medical practitioners, and of dispensing medicines in all parts of the British dominions: provided, first, the course of education required by these institutions or boards be not in any case less, and in the cases hereinafter provided be superior, in extent and duration, to that which shall be determined on as necessary for obtaining a licence; and, second, that the examining boards of these institutions be so constituted, as to afford a sufficient security that the members of whom they are composed possess the qualifications necessary to fit them for ascertaining, by examination, the proficiency of candidates.

5. That the time to be occupied in the minimum course of education above mentioned, at a university or recognized school, should not be less than twenty-seven months, in which should be included three winter sessions of six months' duration each; and that the minimum age of the candidate for a diploma or licence may be advantageously fixed at twenty-one.

7. That apprenticeship should not necessarily form a part of this education; but that those who have not been apprentices should be required to bring proof of having acquired a knowledge of practical pharmacy in a laboratory or an apothecary's shop; and of having had opportunities of witnessing the treatment of diseases, for a period of not less than six months, as pupils to practitioners in dispensaries, or in public hospitals receiving out-patients, or as pupils to regularly-licensed private practitioners.

8. That evidence should likewise be required from candidates for licences, who have not previously obtained the degree of A.B. or A.M., of their possessing an adequate acquaintance with Latin, and of their having received instruction in the elements of mathematics, and in natural philosophy; and that it is highly desirable these branches should be studied previously to commencing the professional education.

9. That strict sessional examinations should be enforced on all students following their course of study in universities or schools of medicine in Great Britain or Ireland, at the close of each winter and summer course; and that a record should be kept of the result of the examinations, to be produced at the final examinations for degrees and licences.

10. That the final examination for the licence to practise should be divided into at least two parts, to be held on different days; and that, in Edinburgh, these examinations may be advantageously conducted by a joint board of Fellows of the Royal Colleges of Physicians and Surgeons.

11. That no person ought to obtain a licence to act as a surgeon who has not gone through a course of education at least equal in duration and extent to that laid down in the minimum schedule of education above stated, and undergone examination before a competent board.

12. That all persons having obtained a licence to practise surgery, in conformity with the above conditions, should be entitled to act as general medical practitioners, and to dispense medicines in all parts of the British dominions, provided they shall have been examined by a competent board or boards, on all the branches of education specified in the curriculum above stated.

13. That the course of study and the examinations for any degree in medicine granted by a university, ought to comprehend all the branches of knowledge stated above, and to imply a more extended education than is prescribed for the general medical practitioner.

14. That, on the other hand, the education for a university medical degree, should not be raised so high, by legislative enactment, above what is required of the general practitioner, as to limit, injuriously for the public, the number of those persons who, in order to obtain the honour, may be induced to take a fuller course than is necessary for a simple licence.

15. That the time to be occupied in the course of education, required for any medical degree from a university, should not be less than thirty-three months, in which should be included four winter sessions of six months' duration each; and that the minimum age of the candidate for such a degree may be advantageously fixed at twenty-two.

16. That the superiority of university medical degrees should be further secured by the course of study required for it, embracing additional branches of science connected with medicine, and by enjoining repeated attendance on the more important departments.

17. That a certain portion of the study qualifying for the honour of a medical degree should be prosecuted in some university which grants that degree, and that any such university ought to insist on attendance therein during a winter session, as preliminary and requisite to examination.

18. That all persons having obtained a medical degree from a university, in conformity with the above conditions, should be entitled to act as general medical practitioners, and to dispense medicines in all parts of the British dominions.

19. That the lectures of all teachers should be recognized as qualifying for the licence of general practitioner who are professors in universities, or fellows of the Royal Colleges of Physicians or of Surgeons in England, Scotland, or Ireland, or of the Faculty of Physicians and Surgeons of Glasgow, and who shall have been specially recognized as teachers by the bodies to which they belong, after they shall have been satisfied, by examination or otherwise, of their being duly qualified to teach.

20. That all other teachers, before their lectures are admitted to the same privilege, should, besides being in possession of a medical degree from a university, or a diploma or licence from one of the Royal Colleges of Physicians or of Surgeons, or of the Faculty of Physicians and Surgeons of Glasgow, or other legally-constituted licensing body, undergo an examination on the branch they propose to teach.

23. That the lectures of teachers, not being professors in universities, but qualified as above stated, ought to be recognized in such extra academical education as may be allowed to qualify for university degrees.

24. That professors and other recognized teachers ought to give courses on the respective subjects of their lectures, of such extent and duration as may be deemed sufficient by the legislature to embrace the full consideration of these subjects.

25. That the lectures of no professor or lecturer who teaches within the same year more than one of the branches required, ought to be recognized; but that, in reference to this regulation, anatomy with practical anatomy, and chemistry with practical chemistry, might be considered as one branch respectively; while clinical medicine and clinical surgery might be taught, in addition to any of the other branches, by professors, physicians, and surgeons, to whom is intrusted the charge of recognized hospitals.

26. That provision should be made in regard to chemists and druggists for their being found sufficiently qualified to compound, prepare, and dispense medicines; and that no persons ought to obtain licences to act as such who have not, first, attended at least one full course of lectures on each of the following subjects, viz. chemistry, botany, and materia medica, and a course of practical chemistry, by recognized teachers; second, been employed for three years in compounding and preparing medicines under a licensed general practitioner, or licensed chemist and druggist; and, third, attained the age of twenty-one years.

27. That, previously to obtaining such licences, the candidates should undergo an examination, before a competent board, on chemistry, botany, and materia medica, and as to their knowledge of the Latin language, and of the forms of prescription.

28. That those persons only who have gone through such education and examination, should be entitled to the name of licensed or approved chemists and druggists, or to such other designation as may imply their qualification; that the licence granted to them should infer no right to exercise the duties of general practitioner, but should entitle them to act as chemists and druggists in any part of the British dominions.

---

III. *Extract from "The Petition of a Meeting of Physicians, Surgeons, and Apothecaries, practising in Ulster, convened by public advertisement, and held in the Belfast Hospital on Wednesday, the 24th of January,* 1838."

That your petitioners humbly and respectfully beg to submit the annexed outline of a plan for the better regulation and government of the Medical Profession in the British dominions, to the consideration and wisdom of your Honorable House; feeling assured that such outline will be fully sustained by the evidence taken before the Committee of your Honorable House; and if adopted, will tend materially to lessen or remove altogether the anomalies, abuses, and mischief, at present existing; while at the same time, it will improve and elevate a profession whose high and peculiar characteristic is to minister to and alleviate the sufferings of mankind.

1st. That a Central Board of Examiners be formed in each of the metropolitan cities—in London, Dublin, and Edinburgh, who shall possess the power of conferring a diploma in medicine, which will entitle the possessor to practise in any or all of the branches, and in any part of the British dominions; that the persons composing these boards be selected from the most distinguished members of the profession in the three kingdoms; shall receive a *stated* annual salary for their services as examiners, and be totally unconnected with any medical school; that they be authorized to frame ONE *curriculum of education*, on an enlarged and scientific scale, for the entire medical profession, which all medical teachers and students shall be strictly bound to observe.

2d. That they be intrusted with the power of recognizing medical schools and teachers; and of punishing, on summary jurisdiction, all persons who presume to teach or practise illegally the profession in any one of its departments. Further, that they be instructed to frame ONE *Pharmacopœia* for the whole empire; and thus remove the inconveniences and risk at present so generally experienced by a want of uniformity in the preparation of several most important and dangerous remedies.

3d. That the existing Universities, Colleges, and Halls be deprived merely of the privilege of conferring medical diplomas, and that they be permitted to exist as schools; retaining all the funds, museums, and other properties they may be already possessed of.*

---

IV. *Extracts from a Statement made to the Home Secretary, by a Deputation from the British Medical Association, June* 2, 1838.

Some of the evils under which the profession labours were briefly enumerated to his Lordship.

First. There were the unreformed medical corporations, eight in number, namely, 1, the Royal College of Physicians; 2, the Royal College of Surgeons;

* Lancet, June 23, 1838.

3, the Society of Apothecaries, in London: 4, the Royal College of Physicians, and 5, the Royal College of Surgeons, in Edinburgh: 6, the College of Physicians; 7, the Royal College of Surgeons; and 8, the Apothecaries' Company, in Dublin: which are evils in themselves, inasmuch as they are nearly all obnoxious to the abuses and defects of the unreformed municipal corporations, which no party now dares to defend.

Secondly. There were the councils, or governing bodies, of those corporate institutions, which are self-elective or self-perpetuating, and the members of which are elected for life.

Thirdly. There was the manner in which the proceedings of those corporate bodies were carried on, than which nothing could be more inconsistent with the principles of the free constitution of this country, inasmuch as they were always carried on *in secret;* and inasmuch, also, as a full statement of the *accounts,* or of the appropriation of the money which has been levied on the members, and has accumulated, is never published.

Fourthly. There was the irresponsibility of those governing bodies, whose councillors considered themselves as being neither amenable to the members at large of their own body, nor to the public, nor to the government of the country; the injurious effect of which practice was to be found in the working of the innumerable by-laws they had each of them (like the rest of the corporations now reformed) enacted. Those by-laws prescribed onerous injunctions on the meritorious student, levied money in hospitals and schools, and promoted private views at the public expense, whereby medical knowledge and medical improvement were checked instead of being promoted.*

---

V. *Extract from a Letter of* Dr. Webster, *President of the British Medical Association, addressed to the Secretary of the Glasgow Medical Association. July* 3, 1838.

The prominent object of the British Medical Association is, 1st. The establishment of a "National Faculty of Medicine," (of which a branch might exist in each capital,) which should comprehend all the legally-qualified practitioners in the kingdom. 2dly. That a senatus or representative council be elected by the faculty, which shall manage the affairs of the profession, and regulate all matters relating to the education, examination, and admission of future members. 3dly. That the members of the faculty shall all possess the same rights, privileges, immunities, and be able to practise in all parts of her Majesty's dominions without let or hinderance. 4th. That the members of the faculty be recognized by the title of Doctor; or they might be divided into two classes, Doctor in Medicine and Doctor in Surgery; and that they shall be at liberty to practise the whole or any branch of the healing art, and write prescriptions only, or dispense their own medicines, as may be found most eligible or convenient. 5thly. That chemists and druggists shall be examined as to their knowledge of chemistry, materia medica, and pharmacy; and be licensed to dispense medicines only, but not to practise any branch of the profession. 6thly. That there be a British Pharmacopœia for the whole empire. 7thly. That all the members of the faculty be registered; and that any person practising without being so registered or holding a diploma or licence from the senatus, shall be summarily punished.†

---

VI. *Extract from a "Report of the Medical and Surgical Society of Newcastle-upon-Tyne, dated Nov.* 27, 1838."

There are in the United Kingdom of Great Britain and Ireland not fewer than sixteen corporations having power to grant degrees in medicine and surgery, and differing essentially in the extent and duration of the curricula they enjoin. The Society is of opinion that all of these might be advantageously superseded by one institution being placed at the head of the profession in each division of the empire, whose privileges should be reciprocal, and whose executive officers, elected by the members at large, should hold periodical conferences, for the pur-

* Lancet, June 16, 1838. † Ibid. July 28, 1839.

pose of establishing uniformity of operation; that such institutions should have entire control over education, and the granting of degrees and licences to practise, together with all other matters relating to the medical profession; that the course of instruction and test of qualification should be the same in each; that, from one or other of them, all persons engaged in the practice of medicine and surgery should be required to possess a diploma or licence; that, to individuals thus authorized, the law should extend a suitable protection; and that proper measures should be enforced for the suppression of unqualified practitioners.

The Society would suggest, as a means of effecting the last-named object, that every person, before commencing practice in any town or other locality, should be required to obtain a certificate from a magistrate, giving him permission to that effect, which should be granted on the production of satisfactory testimonials of qualification, and that, after having been thus authorized, his name should be duly registered. Persons presuming to practise, whose names have not been so registered, should be subjected to a penalty on summary conviction before a justice of the peace.

The rapid progress of science during the present century, in conjunction with increased facilities for the attainment of medical and surgical knowledge, have fully proved that any attempt to constitute an arbitrary division of diseases, and to consign the treatment of them to different classes of practitioners, according as they affect the external or internal parts of the body, is not only unscientific, but impracticable; and, as the physician and the surgeon must be guided by similar principles in combating disease, whether involving the surface or the interior of the human frame, the education of all practitioners should, in the opinion of this Society, be regulated by one common standard. Those distinctions in rank which have hitherto subsisted (not, perhaps, without good effect,) would thus be rendered unnecessary, since there could be no longer any rational ground for separating into different grades men who would be identified not less in education than in the nature and object of their pursuits. Such uniformity, if established in this as in other countries, would place practitioners, whether of medicine or of surgery, on an equal footing; but would not, in the least degree, prevent individuals devoting their energies to the prosecution of any particular department of professional duty, which inclination or other circumstances, might lead them to adopt in preference to another.

The task of preparing the medicines prescribed by the general practitioner, devolves, almost universally, on the apprentice of the latter. That material benefit might accrue from a well-devised scheme of pupilage, there can be no question; but apprenticeships, as at present conducted, have ever been productive of unhappy results; and in no respect does the unfavorable tendency of this system appear more conspicuous than when viewed as an instrument for executing the responsible duty of dispensing. The abolition of apprenticeships, so far, at least, as this department is concerned, would be highly expedient. The Society is of opinion, that a charge so important might, with greater safety, be confided to an apothecary or dispenser, who had been examined in pharmacy, and had obtained a specific licence for the purpose in question. Such substitute for the apprentice would, the Society believes, be most desirable, not less for the comfort and convenience of the practitioner than for the welfare and security of his patients.

This proposition, if acted upon, might have an additional good effect in terminating the absurd method by which at present the majority of general practitioners seek to be remunerated, viz., by a profit on the medicines they supply.

The foregoing representations suggest the desirableness of obtaining,—

1. An improved system of education.
2. A more efficient method of examination.
3. One governing body to preside over the whole profession in England, Scotland, and Ireland.
4. Uniformity of education and of grade among practitioners.
5. Adequate protection for legally-authorized practitioners.
6. The prevention of unqualified persons.
7. The suppression of quackery.

8. The separation of the practitioner and the dispenser in the same individual.
9. The abolition of apprenticeships as at present constituted.
10. The institution of licensed dispensers.

---

VII. *Resolutions agreed to at a Meeting of the Royal College of Surgeons in Ireland, held on Monday, April* 22, 1839.

1. That the College are willing and anxious to adopt such steps as may be considered practicable and expedient, having for their object the incorporation of the whole body of practitioners in Ireland into a firm and powerful union.

2. That, in effecting such a measure, the College conceive that those only can be properly considered as medical practitioners who pursue the healing art as a profession; and that, consequently, pèrsons following the business of retail druggists ought not to be admissible into the proposed union.

3. That in making this restriction, however, the College by no means wish to undervalue the occupation of a druggist or apothecary; but that they consider its pursuit by an individual, who at the same time practises medicine, to be alike injurious to the public and to the parties concerned.

4. That the College are prepared to go the length of seeking a new charter of incorporation, if the proposed union cannot be satisfactorily effected by any other mode.

5. That, inasmuch as some difference of opinion may at present exist, as to the mode of effecting the objects referred to in the resolutions now presented, the College conceive that a general congress of the profession ought to be immediately held; and they now declare that they will cordially assist in the proceedings of such congress, if its assemblage be thought advisable by the mass of the practitioners of Ireland.

6. That it is the opinion of the College that this union of the profession should include both physicians and surgeons, with equal privileges.

7. That, in order to carry into effect the suggestions contained in the report now agreed to, it is resolved, That the College, seeing the utility of a union of all the physicians and surgeons of Ireland, not engaged in the practice of pharmacy, willingly consents to become the centre of such a union, and to afford every facility in its power for the attainment of the important objects contemplated by those who have proposed it.

8. That, as the efficiency of such a union depends upon its being established upon just, sound, and liberal principles, deputies should be sent from every county in Ireland, to represent the opinions and wishes of their respective constituents, or those who are unable to attend, at each general meeting of the society.

9. That the first meeting of a society thus formed, which may be aptly termed "The Medical Union of Ireland," shall be holden in this College on the 29th day of May next, to which all the physicians and surgeons of Ireland, not practising as apothecaries, shall be invited by public notice in the newspapers to attend.

10. That any officers appointed to carry these purposes into effect shall be merely esteemed *pro tempore* until arrangements are made by the first general meeting. That the committee be authorized to give notice by advertisement of these resolutions, and make such arrangements as may be necessary to carry them into effect.

11. That the proposed meeting of the profession is to be held for the purpose of determining upon the best means of forming a union of the profession, and not for the purpose of considering whether such a union is or is not expedient.

12. That the president and secretaries, with the committee of correspondence, be deputed to meet the President and Fellows of the College of Physicians to submit the foregoing resolutions to them, and ask the co-operation of their College.

---

VIII. *Resolutions passed at the Meeting of the Physicians and Surgeons of Ireland, called in accordance with the foregoing Resolutions, and held in Dublin, on Wednesday, the* 29*th May,* 1839, *partly at the Royal College of Surgeons, and partly (owing to want of room in the former) at the Theatre.*

1. That we, the physicians and surgeons of Ireland, having expended large sums of money and much time and labour in the acquisition of professional knowledge, and many of us being engaged in the performance of important duties to the public, feel that it is not unreasonable for us to expect from the government and the legislature protection equal to that afforded to the members of other liberal professions.

2. That it is the opinion of this meeting that we are not protected, inasmuch as while we have voluntarily submitted to a lengthened course of medical study, and trying examinations; and while we have contributed large sums to the revenue in the form of stamp duties, yet our services are constantly enforced in the administration of public justice without any or with insufficient provision being made for our remuneration.

3. That it is the opinion of this meeting that the cause of all these evils is to be traced to the existence of divisions and separate interests among the members of the medical profession; and that their effectual remedy is to be sought for in a permanent union.

4. That the true basis of such union should be a similarity of studies, pursuits, and interests; and that we, the physicians and surgeons of Ireland now assembled, do declare that medicine and surgery are one science and one profession, and that the separate practice of certain departments by distinct individuals is merely an expedient division of labour, having no reference to education or to general professional interests.

5. That it is therefore our opinion that a legislative measure should be sought for by us to unite the medical profession of Ireland into a corporation upon such principles as shall constitute them one national faculty, and thereby identify in feeling and interests the great mass of provincial practitioners with their metropolitan brethren.

6. That, to promote so desirable an end, steps be at once taken to form district medical associations throughout Ireland, which shall be composed of all practitioners in medicine holding degrees or diplomas in medicine or surgery from any of the colleges, corporations, or universities, at present legally authorized to grant the same, who are of irreproachable moral and professional character; and who are not engaged in the business of retailing drugs, or compounding the prescriptions of others.

7. That the members of such district associations shall elect from among themselves a president, vice-president, secretary, and committee; that they shall have the power to nominate delegates to represent their interests at the general meetings of the profession, and that the duties of such associations shall be to obtain local information regarding all matters of medical police; to settle disputes among their own members; to watch over their local interests, and to communicate with a central metropolitan council.

8. That the following are the principles which should be held in view in constructing the constitution of the new College, which it is proposed to establish: *a.* Fundamental regulations with regard to the education of all persons proposing to enter the profession of medicine, to be permanently established. *b.* Compliance with such fundamental regulations to be rewarded with the licence of the college, conferring free and equal right to all professional practice, office, and emoluments. *c.* All persons so licensed to be enrolled as members of the corporation, upon the expiration of a certain period of probation, provided only they can produce evidence of an irreproachable moral and professional character. *d.* Every person so enrolled, to be thenceforward free to vote and act in the college in every corporate capacity whatsoever. *e.* A general meeting of the whole college to be held on the last Wednesday in May in each year (being the anniversary of the present congress) to which it may be lawful for the district associations to send representatives, the business of such meeting being to elect a president, secretaries, and an executive council, which shall carry on the government of the college during the ensuing year; but shall at all times sit, deliberate, and vote with open doors. *f.* A general meeting to be called at any time by the council, or upon a requisition signed by at least twenty members.

The title of this college shall be the Royal College of Medicine and Surgery in

Ireland, and it shall be to all intents and purposes a union of the two branches of medicine and surgery into one faculty.

9. That at the first formation of the new College, all persons holding degrees or diplomas in medicine or surgery from any of the Colleges or Universities at present legally authorized to grant the same, who have been five years in the practice of their profession, not following the business or profession of a retail druggist or apothecary, and who can produce evidence of irreproachable moral and professional character, shall be enrolled as members of the corporation, upon payment of a sum not exceeding 20 guineas.

10. That persons similarly circumstanced, but who have not been five years in practice, shall, on the payment of a like sum, not exceeding 20 guineas, be enrolled as licentiates of the college, and shall be held qualified to be enrolled as members of the corporation, upon the completion of the full term of five years from the date of their original degree or diploma, without further expense.

11. That members and licentiates of the College of Surgeons, and fellows and licentiates of the College of Physicians (provided the latter body join the union) shall be exempted from any such payment, they having already paid large sums, and now possessing exclusive rights in the present colleges.

12. That persons now enrolling their names, and pledging themselves to support the foregoing resolutions, shall, together with the members and licentiates of the College of Surgeons, and of the College of Physicians (in case that body join in the union) constitute an association to continue in existence until the new charter or act of incorporation shall have been obtained, and that the provisional body so framed shall be constituted as to its officers, meetings, &c., as nearly as possible upon the principles already laid down for the constitution of the new college

13. That a provisional council, secretaries, and president be now elected, and that the business of organization be at once commenced.

14. That Richard Carmichael, Esq. be president of the provisional council, that Dr. Maunsell shall be secretary, and that the council shall consist of the committee of correspondence of the College of Surgeons, with the deputies of the local associations now existing, or hereafter to be appointed, with power to add to their number, and to communicate with medical associations in the sister kingdoms.

15. That it is our firm conviction that the interests of the public, and of the medical profession, require that encouragement should be given to a class of scientific apothecaries, whose time and attention would be exclusively devoted to the preparation and compounding of medicines, and who would thus have an opportunity of raising the profession of pharmacy from its present degraded condition in Ireland to a level with that which it occupies in France and Germany; and that we think such encouragement would be best afforded by the establishment of a College of Pharmacy; by the prevention of medical practitioners from keeping shops for the sale of drugs, or compounding the prescriptions of others, and by affording to regularly-educated apothecaries protection, by giving them an exclusive right of dealing in medicinal articles, and such other advantages as could fairly be granted to them by the legislature.

16. That we disclaim all intention of interfering by any of our proceedings with vested rights now enjoyed by any individuals.

17. That the establishment of a relief and widows' fund be recommended to the attention of the provisional council.

18. That the best thanks of the meeting be given to Mr. Carmichael for his admirable conduct in the chair.

---

REMARKS.

We have been waiting, quarter after quarter, we may indeed say year after year, for the publication of the Report of the Parliamentary Committee of Enquiry into the state of the medical profession, in order that we might call the attention of our readers to a subject of such surpassing interest, and state our own opinions and views respecting it. As, however, such Report has not made its appearance, and, we fear, is not likely soon to do so, and as, consequently, the prospect of any legislative enactment to be derived from this source seems nowise probable, we are compelled by the magnitude and urgency of the question at issue, to enter upon it with such help as we are able to derive from the exertions and demonstrations made by the profession itself since the institution of that enquiry. On the present occasion, however, it is our purpose merely to open the subject; reserving for another and we hope an early opportunity the ample discussion which it requires, and to which it is so well entitled.

The preceding documents, and we might have added others of a similar character from the same sources, sufficiently show how general and deep is the impression among the members of the profession of the necessity of medical reform. We have reason to believe that this impression will be still further strengthened and extended by the proceedings of the Provincial Medical and Surgical Association, at the next meeting of the members, to be held at Liverpool on the 24th of the present month. We regret that we are not at liberty to publish the Report of a Committee appointed by the Association for the purpose of watching over the interests of the profession, which will be laid before the members at this meeting. We may, however, state that this Report strongly recommends the same consolidation and union advocated in the preceding documents; and differs only from these in giving the subject of EDUCATION as the basis of the reform, a more prominent place and character. It is because we so perfectly accord in this view of the subject, that we have united, under one head, in the preceding pages, the documents relating exclusively to education, and those bearing chiefly on the external arrangements of the profession and the privileges of its members. It must be manifest to every one qualified to judge, and who takes a proper view of the subject, that a sufficient and well-arranged education, as well preliminary as professional, must lie at the root, and indeed must form the indispensable basis of all rational and fructifying reform. This truth was fully admitted by several of the distinguished speakers at the great Dublin meeting, a circumstance which we are the more anxious to point out, as, although it is recognized in the resolutions, it certainly occupies a less prominent place in them than is desirable. "It was proposed (Dr. Maunsell said) that the road of entrance to the medical profession should be that of education alone; that there should be the highest possible standard of education established; and under no other circumstances would any man be permitted to enter. This was one of the grand objects of the union, and one, which necessary as it was to the public, and the medical profession, could yet never be effected so long as sixteen or seventeen distinct and rival corporations existed and competed with each other, not in generous efforts to raise the standard of medical education, but in a Dutch auction, as to which of them should give the licence to practise on the cheapest and easiest terms." "The resolutions (said Dr. Bullen) called upon the legislature to enable them to obtain an act—for what? Why, to effect medical educational reform: for the first great principle upon which the act must be framed, must be to elevate the medical profession to the highest standard of eminence. It was only by elevating the masses that they could raise the units, and there was no man whose interest would not be advanced by every point in which they could raise the general medical education of the practitioners of Ireland." It is certainly true that many advocates of medical reform who have evinced a strong desire to regulate the outer and grosser mechanism of the profession, have paid but slight regard to the finer, less visible, but much more important springs within. But this, unquestionably, is beginning reform at the wrong end. We, on the other hand, are so impressed with the paramount importance of the education, that we desire to see it take precedence in all deliberations respecting the improvement of our profession; and this must be our excuse for confining our remarks, on the present occasion, so much to its consideration.

It is but justice to almost all our examining bodies to state, that, of late years, they have much raised the standard of qualification in the candidates for their licences, by insisting on more extended studies. In the "Propositions" from Edinburgh, given above, we see further indications of a greatly improved system of education. It is, however, in the document placed first in our series, the "Regulations" of the University of London, that we recognize, for the first time, all the essential elements of a complete medical education; and we do not hesitate to avow our belief, that whatever other improvements may be the result of the medical agitation now on foot in Great Britain and Ireland, they will be imperfect unless they contain provisions for enforcing an education, preliminary and professional, as liberal, at least, if not precisely the same. Indeed, we do not know that much more is wanting to satisfy intelligent reformers than the establishment in each of the other divisions of the empire of an institution like the University of London, only constructed on a more enlarged and liberal basis and endowed with higher powers and privileges. The first great desideratum for the improvement of the profession is, unquestionably, to have established, by legislative enactment, a *minimum* of preliminary and medical education, which shall duly qualify for successful practice; and it appears to us that the course of instruction prescribed in the regulations of the University of London, is a near approximation to this. Without being so complete, perhaps, as is desirable, it is, we think, as good as can be obtained under the defective system of teaching, which still prevails in our schools generally. In our opinion, the University requires nothing in their "Examinations in Arts," which may not easily be acquired by a youth of ordinary abilities, of seventeen or eighteen years of age; and we believe that the amount of professional knowledge required of the candidate for the first medical degree may be easily obtained during the five years subsequent to such preliminary training.*

Now, let us suppose that the London University, in addition to its present powers, were legally constituted the sole examining and licensing body for England; that similar bodies were established in Edinburgh and Dublin, with like authority for Scotland and Ireland respectively; and that the degree of *Bachelor of Medicine* were made the essential qualification for the practice of medicine and surgery; that, in fact, the Bachelors of Medicine, (or by whatever other name they might be recognized) should constitute the class of *General Practitioners;* we have thus, at once, the elements of the only reform that can be required or ought to be received by the members of an enlightened profession—a reform based on improved education, leading to improved knowledge and skill, and enhancing professional respectability and honour. According to this arrangement, the Bachelors of Medicine being the general practitioners, the Doctors of Medicine would constitute the higher ranks of the profession, and fill the offices now usually filled by physicians and surgeons, (or *pure* surgeons, as they are sometimes called.)

This arrangement, while recognizing the unity of the profession and the right of all to practise any of its branches, would still admit of those divisions into classes and those differences of rank, the convenience and utility of which have been sanctioned by experience. The equality of all the members of the profession would only be broken by the legitimate claims of superior talents, higher studies, or more enlarged experience.

Such a great change in the state of the medical profession as that contemplated in these observations and in the documents which precede, cannot, of course, be effected without the interference of the legislature; and is, no doubt, attended with great difficulties. But the necessity of reform is now so apparent, and the voice of the profession has been at length raised with such earnestness and energy, that government, we think, cannot help giving the subject their early and serious consideration. The simple fact, stated in one of the preceding documents, that

* The only doubts we entertain on the subject are—1. Whether the prescribed education may not be found too expensive for the means of the present class of medical students; 2. Whether the means of the inhabitants of certain localities, particularly in country districts, may not be found inadequate to supply a fair remuneration to men who have had so expensive an education. It is to be recollected, however, that the abolition of apprenticeships, which must form part of the plan of reform, would cut off an important source of expenditure of money, as well as time.

It is not our intention, at present, to notice in detail, either of the two curricula given among the preceding documents; but we cannot pass by two most important improvements. In the Edinburgh "Propositions," it is recommended, "that a portion of the study should be allowed to be prosecuted under extra-academical teachers;" and it forms a characteristic of the London Curriculum, that the students have, in a very considerable degree, the option of choosing the particular Lectures they shall attend. This regulation, if generally adopted, will have the effect of removing all bad teachers from Universities and medical schools, by the very simple process of leaving their benches empty.

there exist at this moment sixteen different examining and licensing bodies in Great Britain, each requiring a different amount of education, is sufficient to show the absolute necessity of legislative interference to put an end to a state of things which is little less than disgraceful in a civilized country. To carry such a scheme into effect much time, much patience and labour on the part of its advocates, and not a few sacrifices on the part of many of the existing public bodies, will be requisite; but we, for our parts, see nothing impracticable in it; and we regard the great ends to be obtained by its successful prosecution as justly claiming the utmost exertion of every individual who approves of it, and justifying the many and great sacrifices necessary for its accomplishment.

To our brethren in England and Scotland we would hold up the proceedings of their fellow-labourers in Ireland as worthy of all approbation and imitation. In their operations hitherto, crowned by the great congressional meeting in Dublin, they have shown so much zeal, energy, unanimity, and disinterestedness, as not only deserves but must obtain success. If they proceed, as we doubt not they will, in the course they have begun, they may depend on the warm sympathy and active co-operation of their brethren in England. Reform, to be successful in any one division of the empire, must be claimed by all—it must be general, not partial; and we are glad to observe that this is the view taken of it by the meeting in Dublin. "Although," said Dr. Bullen, "they attempted to found a national Irish faculty, they could not stand insulated as a national body. They had the medical institutions of England and Scotland, and they never could, and never ought, to attempt to carry any measure which would exclude the regularly-educated graduates of other British schools. It would ill become them, having entered within those walls, that their first step should be to carry out that principle of exclusion which they so unanimously condemned. Therefore, the movement that day was not exclusively an Irish one, but one that would be responded to by all the schools of England and Scotland. The principle which the Irish and other schools advocated, was to elevate and advance the medical profession by elevating and advancing medical education. It might be apprehended that they were only endeavouring to cloak another monopoly by the proceeding which they had undertaken, and the only way to avoid such a suspicion was to put forward in every way that they could, that the only test required of a man's competency was, that when the individual was to receive the degree giving him a licence to practise any of the departments of the medical profession, that degree should be a *bonâ fide* test of his intellectual capacity and educational acquirements. If they adopted this principle, no government that valued education in man or recognized it as important to the great interests of society could refrain conscientiously and consistently from uniting with them in saying that a cry for medical educational reform had been raised which should be carried out."

But, moreover, we think it essential to the success of this great measure, not merely that the reform shall be founded on an improved education and higher qualifications in the candidates for licences to practise, but that the body which regulates the education, tests its results, and grants the licences, shall be restricted to the exercise of these duties, having nothing whatever to do either with the direct communication of instruction to students, or with the conduct or proceedings of candidates after they have received their degrees or licences. Other bodies of course, whether under the name of simple schools or chartered colleges, must exist for the purpose of imparting instruction; others, again, for purposes of a scientific or merely professional kind, like our present colleges of physicians and surgeons, the college of pharmacy proposed in the Dublin resolutions, &c. &c.; but the all-important institution, and the establishment of which must be looked upon as the great object of the efforts now making, is, as Dr. Henry well expressed it at the Dublin meeting, "a University rather than a College,—not a teaching, but a licensing body, that should have a relation to the existing colleges, somewhat similar to that which exists between the university of Oxford and its component colleges." It is to the establishment of such a body as this in Dublin and Edinburgh, with exclusive powers of regulating medical education, examining candidates, granting degrees, and licensing to practise, that the chief efforts of the profession should, we conceive, be now directed in Ireland and Scotland; and it is for the addition of the exclusive licensing power to those powers already possessed by the new university of London that the reformers of England should mainly strive. For conferring these powers on the three metropolitan institutions an act of the legislature is essential; all the other arrangements of the profession as a body might, we think, be carried into effect afterwards by the efforts of the members themselves, or, at least, without any legislative enactment. In our view of the subject, all authority of the universities should cease with the act of licensing, and then only should the authority of such bodies as colleges of physicians and surgeons begin. The objects of such colleges should be altogether different from those of the universities. They would be reunions of the members of the profession for scientific and professional purposes, to which all ought to have equal access, and in which all should have the same privileges. They would constitute, as it were, the parliament of the profession in their respective divisions of the kingdom. They would be the guardians of the rights of the members, as well as of their honour and respectability; they would promote the cultivation of science by their countenance, by their funds, and by their honorary distinctions; they would be the centre and medium of professional union; in a word, they would protect and promote, in every way, the interests of a profession composed of men all liberally educated and all possessing an equality of rights. For the better accomplishment of these and many other most important objects, it would, no doubt, be highly desirable that the present colleges of physicians and surgeons should be united into one institution, in each of the three capitals. In these the members might still divide themselves into sections, for the cultivation of different departments of medical science, although it is obvious that the science itself can only be divided into different parts by artificial violence.

